# Ineffective implementation of emergency reduction measures against high concentrations of particulate matter in Seoul, Republic of Korea

**DOI:** 10.1007/s10661-023-11754-0

**Published:** 2023-08-31

**Authors:** Chang-Hoi Ho, Ka-Young Kim

**Affiliations:** School of Earth and Environmental Sciences, Seoul National University, 1 Gwanak-Ro, Gwanak-Gu, Seoul, 08826 Republic of Korea

**Keywords:** Air pollution, Emergency reduction measures, Emission source, Korea, Pollutants, Seoul, Traffic volume, Transboundary transport

## Abstract

Since December 30, 2017, the Seoul Metropolitan Government, Republic of Korea, has been implementing emergency reduction measures (ERMs) restricting the operation of industrial sites, thermal power plants, and vehicles when air quality is expected to deteriorate. ERMs are implemented when the present observed concentration of particulate matter (PM) of aerodynamic diameter less than 2.5 μm (PM_2.5_) and/or the predicted values for the following day exceed a threshold value. In this study, the effectiveness of ERMs was evaluated for 33 days with and 6 days without ERM implementation but where the PM_2.5_ concentration exceeded the threshold value, until March 15, 2021. Of the 33 days of ERM implementation, on 7 days it was executed despite the thresholds not being met. The ERM on these days might have been properly executed because the pre-notice and implementation of ERM might have reduced the local emissions of air pollutants. Our major findings are that even on days of ERM implementation, there were marginal reductions in vehicle traffic, thermal power generation, and industrial emissions. Second, the concentrations of PM_2.5_ and related air pollutants in Seoul were almost unchanged for most ERM implementation episodes. Third, most of the 39 (= 33 + 6) days when the air quality worsened were caused by the transboundary transport of air pollutants from China. In conclusion, it was revealed that the currently executed ERM law is insufficient for effectively reducing PM_2.5_. To achieve the required reductions, it is necessary to undertake stricter policies in Seoul and its neighboring regions.

## Introduction

The Seoul Metropolitan Government has been implementing emergency reduction measures (ERMs) in Seoul, Republic of Korea (hereafter referred to as Korea), on days when extremely high levels of particulate matter (PM) with an aerodynamic diameter less than 2.5 μm (PM_2.5_) are expected to continue for a certain period (KME, 2019). The ERM law was enacted on March 15, 2015; however, it was first enforced on December 30, 2017, after supplementing various associated materials and increasing the accuracy of PM predictions for the next 1 to 2 days. When the ERM law was implemented, strong regulations were imposed on vehicle operations and various sources of air polluting emissions. By reducing, as far as possible, locally generated emissions in the Seoul area, a rapid increase in the concentration of PM_2.5_ can be alleviated, even when air pollutants flow into Seoul from surrounding regions.

Nevertheless, ERM should be executed in a timely manner, because it causes an enormous inconvenience for social and economic activities (Choi et al., 2019b; Yoon, 2019). When ERM was promptly initiated on January 13–18, 2018, it was estimated that PM_2.5_ precursor emissions decreased by 26–49% (Joo et al., 2018). Lee et al. (2019b) suggested that emission amounts could be diminished by ERM, resulting in a 30% decrease in PM_2.5_ concentrations. In many countries, multiple policies have been implemented to decrease short-term PM concentrations when air quality is expected to be extremely poor. In China, odd–even vehicle schemes and a shutdown of emission sources were enforced in the Beijing–Tianjin–Hebei (BTH) region; these actions reduced daily mean PM_2.5_ concentrations by 20–30% (Wang et al., 2017, 2018). In contrast, there are some cases of trivial reduction effects due to significant inflows of air pollutants from neighboring regions (Lee et al., 2013; Oh et al., 2020). Considering that the above-mentioned studies generally reached their conclusions based on case studies, there was a limited ability to generalize the effectiveness of the reduction measures. Moreover, numerical modeling results have been used to artificially adjust emissions without aggregating the observed reductions in traffic volumes and power generation (Ma et al., 2020; Tian et al., 2019).

While a total 33 days of ERM were implemented in a timely manner from the time the law was implemented until March 15, 2021, the number of days of high PM_2.5_ concentration has not significantly decreased (Lee et al., 2019b). For this reason, it is necessary to investigate not only the reduction in local emissions in the Seoul metropolitan region but also other influences from domestic emissions, transboundary transport, and atmospheric circulation (Chang et al., 2021; Lee et al., 2011, 2021; Oh et al., 2015). Under stagnant synoptic environments, locally emitted pollutants might not diffuse to the surroundings (Park et al., 2019), and transboundary transport pollutants can combine with local emissions to react chemically (Kim et al., 2017b; Seo et al., 2017); thus, extremely high concentrations of PMs could last for several days. It should be noted that Korea is located on the windward side of China; thus, the air quality may deteriorate due to the inflow of transboundary air pollutants from China (e.g., Bae et al., 2019; Chang et al., 2021; Kim et al., 2017b, 2016a; Lee et al., 2013). During the period of high PM_2.5_ concentrations in Seoul, the contribution from China was 60–80% (e.g., Choi et al., 2019a; Kim et al., 2018; Koo et al., 2008; Oh et al., 2020).

ERM is a critical national issue; however, no research has quantitatively evaluated the effectiveness of the policy. In this study, the effect of ERM was examined by comparing the PM_2.5_ concentrations for four categories of ERM: proper (ERM-proper), late (ERM-delay), effective or improper (ERM-uncertain), and absent (ERM-miss) implementation. The “Materials and methods” section describes the data and methods used in this study. The “Results and discussion” section analyzes changes in PM_2.5_ concentration and air quality variables before and after the period of ERM for the four categories. The domestic and foreign contributions of PM_2.5_ in the Seoul area are also examined. The results are summarized in the “Conclusion” section.

## Materials and methods

### Air pollutant and meteorological data

The daily mean concentrations of air pollutants—PM_2.5_, carbon monoxide (CO), nitrogen dioxide (NO_2_), and sulfur dioxide (SO_2_)—were obtained from 25 air quality monitoring sites in Seoul during the cold seasons (November through March) of 2017–2021. The concentrations of PM_2.5_, CO, NO_2_, and SO_2_ were measured using beta-ray absorption, non-dispersive infrared, chemiluminescence, and pulse ultraviolet fluorescence methods, respectively (Kim & Lee, 2018; KME & NIER, 2019). The measurement error for PM_2.5_ is 10%, owing to the effects of particle-containing moisture, and 5% for the other gases influenced by moisture interference and/or impurities (a full explanation can be found at http://www.law.go.kr). The daily mean surface air temperature, relative humidity (RH), precipitation, and wind speed and direction in Seoul were obtained from the Automated Synoptic Observing System (ASOS) of the Korea Meteorological Administration (https://data.kma.go.kr). The ASOS station in Seoul (located at 37.57°N, 126.97°E) is the sole synoptic station that represents meteorological conditions over the region.

### Traffic volume data

To examine the immediate response of ERM implementation, the total daily traffic volume was obtained from the Transport Operation and Information Service (TOPIS) (https://topis.seoul.go.kr). TOPIS manages the entire traffic system by collecting data at hourly intervals through unmanned detectors installed downtown; at the city borders; and on bridges, and highways. Here, the traffic volume indicates the number of passing vehicles, both inwards and outwards, detected by the 135 monitoring stations in Seoul (TOPIS, 2020).

### Thermal power generation data

The daily electricity generated from thermal power generation by coal-, LNG-, and oil-fired power plants located in Incheon, Gyeonggi province, and Chungcheong province were analyzed from 2018 to 2021. The thermal power plants in these three regions are located along the western coast close to Seoul and are intensively regulated when ERM is implemented in Seoul. Of the 57 coal-fired power plants nationwide, 34 are situated in these three regions, producing 57% of the domestic thermal power generation, with a cumulative power capacity of 21,340 MW (https://kosis.kr). For the LNG-fired power plants, of the 193 LNG-fired power plants, 137 are located in this same region. The greatest proportion of electricity in Seoul is produced through the operation of LNG-fired power plants that emit a large quantity of air pollutants (Kim et al., 2018; Shim & Hong, 2016). Four of the seven oil power plants nationwide are located in Gyeonggi Province. Thermal power generation data were obtained from the public data portal (https://www.data.go.kr).

### Emissions from large manufacturing companies

The total daily emissions of nitrogen oxides (NO_x_), sulfur oxides (SO_x_), and total suspended particles (TSP) from 25 large manufacturing companies were analyzed to confirm the local emission management of air pollutants in Seoul for the period 2019–2021. All 25 companies emitted NO_x_, while SO_x_ and TSP were emitted by two and six companies, respectively. The air pollutant emission data observed by the tele-monitoring system (TMS) of the stack were obtained from the public data portal, which provides information including types of emission and amounts of pollutant emitted (unit: kg). The TMS constantly measures air pollutants emitted by major industrial emitters through remote automatic sensing equipment to produce data every 5 min and 30 min.

### Particulate source apportionment technology

To calculate the contribution of regional sources to PM_2.5_ concentrations in Seoul, chemical modeling was performed using the Weather Forecasting and Research (WRF) model for meteorology, the Comprehensive Air-Quality Model with extensions (CAMx; ENVIRON, 2013) for chemical fields, and the particulate source apportionment technology (PSAT) for chemical transport (e.g., Ho et al., 2021; Oh et al., 2020). The WRF model domains comprised the master grid (27 km) for East Asia and the nested grid (9 km) for the Korean Peninsula. More detailed information has been provided by Koo et al. (2015, 2018). CAMx was used to simulate the chemical fields using WRF modeling data with fifteen vertical layers. PSAT was used to distribute and track the contribution of primary and secondary PM species in the receptor region by simulating the physical and chemical changes in emissions from source regions (Kim et al., 2017a; Wu et al., 2013). Previous studies (e.g., Choi et al., 2022; Koo et al., 2015; Oh et al., 2020) reported that the CAMx-PSAT model well simulated the contribution of each emission region to the PM concentrations in Seoul. This model allows us to quantitatively estimate the contributions of ten regions in China, the Democratic People’s Republic of Korea, and eight regions in the Republic of Korea. These contributions were expressed as percentages for a total of 20 regions, including Seoul. We then further analyzed the output data obtained from CAMx-PSAT. By multiplying the contribution percentages calculated by CAMx-PSAT with the daily observed PM_2.5_ concentrations in Seoul, we could identify the quantitative changes in PM_2.5_ concentration in Seoul and its corresponding source regions during the implementation period of ERM.

### ERM implementation conditions and constraints

The Seoul Metropolitan Government executes ERM for three scenarios (KME, 2019): (1) the mean concentration of PM_2.5_ on the day exceeds 50 μg m^−3^ and it is expected to exceed 50 μg m^−3^ the next day; (2) an alert or warning of raised PM_2.5_ is issued and the mean PM_2.5_ is expected to exceed 50 μg m^−3^ the next day; and (3) if the mean PM_2.5_ is predicted to exceed 75 μg m^−3^ the next day. In other words, ERM is executed when the PM_2.5_ concentrations remain above 50 μg m^−3^ for two consecutive days or above 75 μg m^−3^ for 1 day.

On the contrary, ERM can be disregarded in three circumstances: (1) ERM is no longer necessary due to abrupt changes in weather circumstances (e.g., strong wind and abundant precipitation), (2) the updated forecast of PM_2.5_, after implementing ERM, is below 35 μg m^−3^ on the following day, and (3) the next day is a national holiday. Once ERM is executed, the mayor of Seoul strictly controls vehicle transportation (e.g., odd–even license plate policy and prohibition of heavy-duty diesel trucks), factory industrial complexes, and massive construction sites. Power plants also need to shorten their operation hours, and there are restrictions on the fuel used.

### Four categories of ERM: proper, delay, uncertain, and miss

Prior to ERM implementation, the policy of the Seoul Metropolitan Government was to decrease the monthly and annual mean air pollution concentrations. However, there were no countermeasures against high concentrations of PM_2.5_ that occurred over a certain day or period. On February 15, 2017, ERM was introduced in Seoul as a pilot measure to reduce significant short-term increases in anticipated air pollutants. It was enacted as a mandatory law and applied nationwide on February 15, 2019 (KME, 2019). In the present study, 33 days of ERM implementation in Seoul were selected during the cold season (November–March) annually before March 2021 (Table 1). Six days when ERM should have been implemented but was not (ERM-miss) were also examined. There were no days in 2018 when ERM was implemented expediently (ERM-proper, 11 days), but the occurrence of correct implementations increased rapidly from February 2019. In contrast, most of the days where implementation was delayed by 1 or 2 days (ERM-delay, 15 days) occurred before February 2019, during the ERM pilot phase. Effective or improper implementation (ERM-uncertain) occurred for 7 days, amounting to no more than 3 days per year. These 7 days were executed despite not meeting the ERM threshold. Note that the days of ERM-uncertain might have been appropriately executed because the prediction and/or implementation of ERM may have significantly reduced the locally emitted air pollutants. Of course, the ERM may also have been implemented incorrectly. We do not know which possibility is correct; therefore, we define this category as uncertain.

**Table 1:** Dates of ERM-proper, ERM-delay, ERM-uncertain, and ERM-miss implementations during the cold seasons (November–March) of 2017–2021 in Seoul, Korea. On the underlined dates, the conditions for ERM implementation were satisfied but not executed

ERM implemented		ERM none implemented	
ERM-proper (11 days)	ERM-delay (15 days)	ERM-uncertain (7 days)	ERM-miss (6 days)
12–30-2017 02–22-2019 03–01-2019 03–02-2019 03–03-2019 03–04-2019 03–05-2019 03–06-2019 12–11-2019 03–11-2021 03–15-2021	01–16-2018 01–17-2018 01–18-2018 03–24-2018 03–25-2018 03–26-2018 03–27-2018 01–12-2019 01–13-2019 01–14-2019 01–15-2019 02–14-2020 02–15-2020 02–13-2021 02–14-2021	01–15-2018 11–07-2018 02–23-2019 03–07-2019 12–10-2019 01–11-2020 03–12-2021	02–24-2018 12–22-2018 02–25-2019 03–28-2019 12–11-2020 02–07-2021

## Results and discussion

The effectiveness of ERM was assessed by examining variations in the factors affecting PM concentrations on the days before and after its implementation. In this section, the time series of air pollutants, meteorological variables, traffic volumes, power generations, emissions from large manufacturing companies, and regional (foreign and/or domestic) PM_2.5_ contributions were analyzed for the four categories of ERM, namely ERM-proper, ERM-delay, ERM-uncertain, and ERM-miss.

### PM_2.5_ and three major air pollutants

**Fig. 1:**
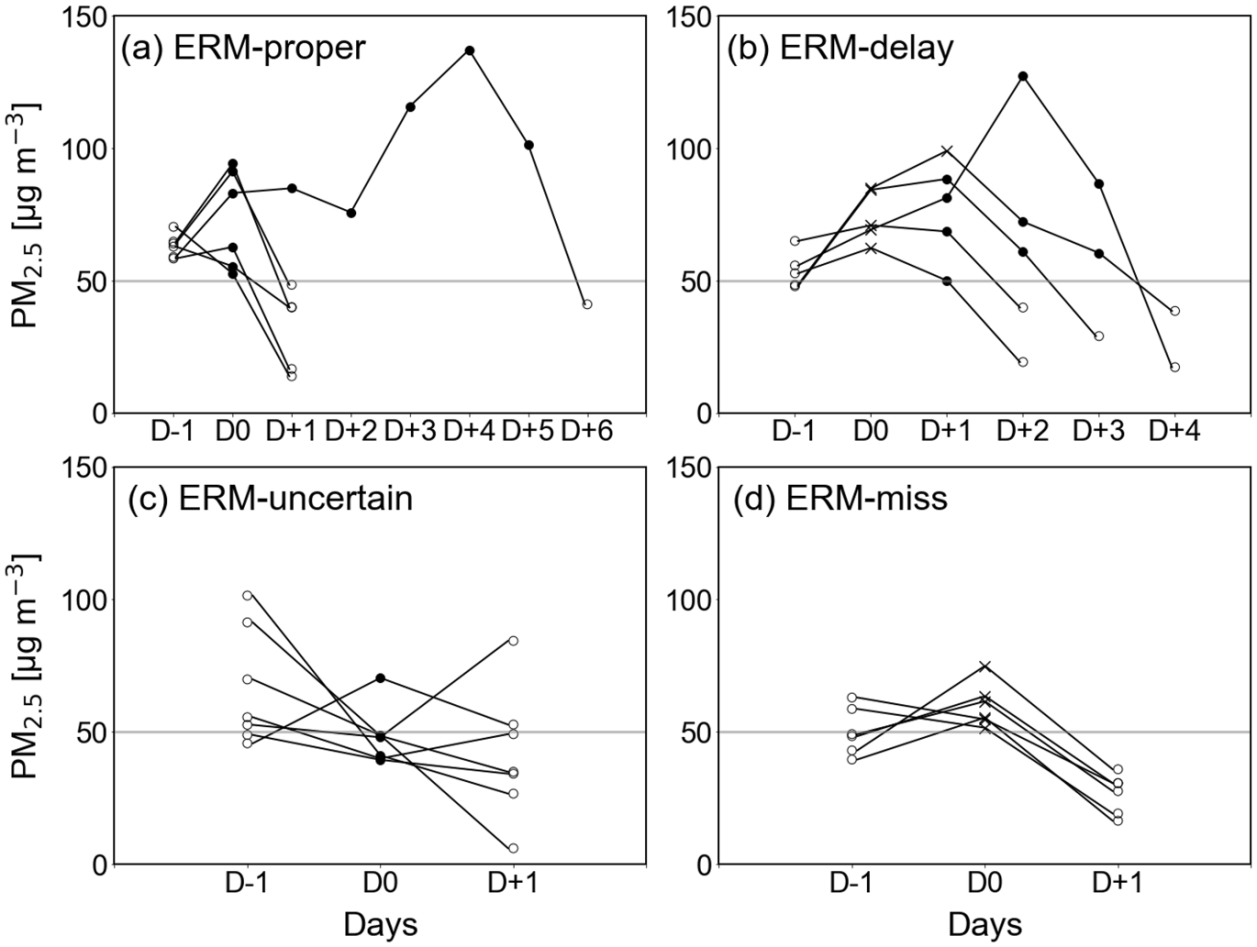
Time series of PM_2.5_ concentrations for the days before and after the first ERM implementation for the particular episode of **a **ERM-proper, **b **ERM-delay, **c **ERM-uncertain, and **d **ERM-miss during the cold seasons (November–March) of 2017–2021 in Seoul, Korea. D0 is the day on which ERM was first implemented or should have been implemented, D − 1 indicates 1 day before the first ERM, and D + 1 to D + 6 indicate 1 to 6 days after the first ERM. Black circles and crosses denote the day the ERM was implemented or failed to be implemented, respectively. The open circles are the days before and after the ERM

Figure 1 displays the time series of PM_2.5_ concentrations for days before and after the first ERM implementation during each episode of the four ERM categories. For the ERM to be executed, as explained in the “Materials and methods” section, the PM_2.5_ concentration should be ≥ 50 μg m^−3^ for two consecutive days or ≥ 75 μg m^−3^ on a single day; therefore, the concentration at D − 1 (the day before the first ERM implementation) was typically greater than 50 μg m^−3^. The ERM law can be implemented for more than 1 day, and these days are considered part of the same ERM episode. As seen in Fig. 1, most ERM implementations span a single day, aside from a single episode of ERM-proper (Fig. 1a) and five episodes of ERM-delay (Fig. 1b). For March 1–6, 2019 (see Table 1), the PM_2.5_ concentration continued to increase even after six ERM-proper implementations. The high concentrations during this episode were mainly attributed to the influence of local atmospheric stagnation along with the transboundary transport of severely polluted air from China (Chang et al., 2021; Lee et al., 2019a).

In the ERM-delay category (Fig. 1b), the duration of PM_2.5_ concentration ≥ 50 μg m^−3^ was 3 to 5 days, which is significantly longer than the other three categories. In this category, ERM was implemented on D + 1 or D + 2 (i.e., 1 to 2 days later than the day when ERM should have been executed), and the PM_2.5_ concentration continued to increase until that point. Even 1 to 2 days after ERM implementation, the concentrations remained over 50 μg m^−3^, and thus the ERM-implemented status was preserved. For the days in the ERM-uncertain category (Fig. 1c), PM_2.5_ concentrations were slightly below the ERM threshold values of 50 μg m^−3^ at D − 1 or D0; thus, it was difficult to determine whether ERM was executed incorrectly. Specifically, PM_2.5_ concentrations less than 50 μg m^−3^ appeared at D0, except for one episode, which may be the effect of ERM implementation, as discussed above. In the ERM-miss category (Fig. 1d), the PM_2.5_ concentration fell below the ERM threshold values within 1 day. On four of six episodes in the ERM-miss category, an alert or warning of PM_2.5_ was issued, and the PM_2.5_, concentration exceeded 50 μg m^−3^ the next day, satisfying the criteria of the ERM implementation. However, even without the ERM, the increase in PM_2.5_ concentration from D − 1 to D0, was not large compared to the other three ERM categories and decreased considerably at D + 1.

**Fig. 2:**
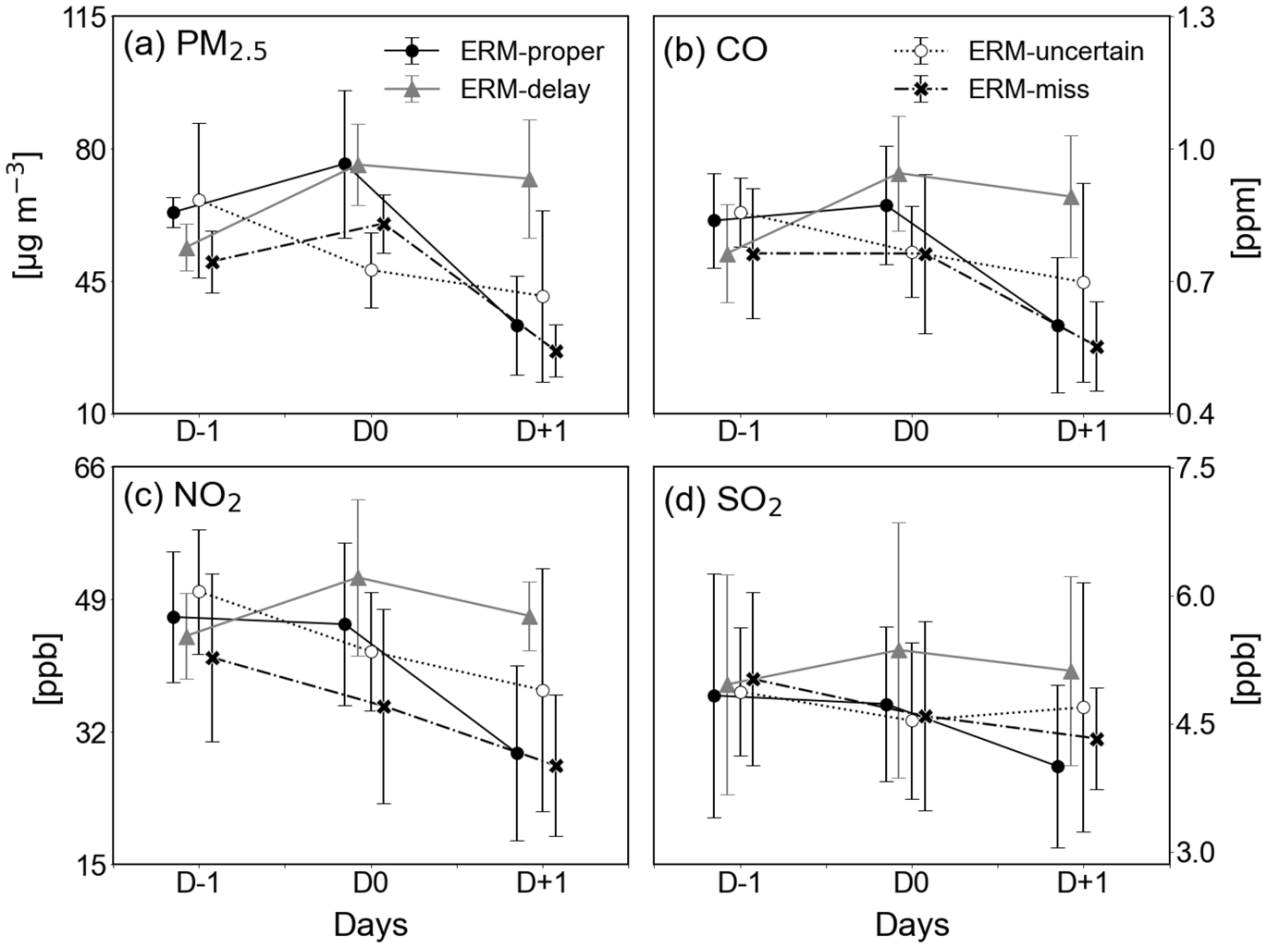
Variations of major air pollutants, **a **PM_2.5_, **b **CO, **c **NO_2_, and **d **SO_2_ at D − 1, D0, and D + 1, obtained by combining the ERM episodes for the four categories. The center line indicates episode mean concentrations of air pollutants and the error bars represent their ± 1 standard deviation

Focusing on the days before and after the first ERM implementation, variations in the concentrations of major air pollutants, such as PM_2.5_, CO, NO_2_, and SO_2_, are shown in Fig. 2. The mean and standard deviation values of the episodes are represented for the four ERM categories. Overall, the changes in all air pollutants exhibited similar features in all categories during the 3 days from D − 1 to D + 1. For the ERM-proper category, the PM_2.5_ concentration increased from D − 1 to D0. However, the concentrations of the other three air pollutants remained relatively constant. In the ERM-delay category, the concentrations of all air pollutants increased dramatically from D − 1 to D0 and remained unchanged at D + 1. It is noted that the ERM was implemented at D + 1 rather than D0 in this category. The forecasters did not expect a sudden and continued increase in pollutants; thus, they may have missed the proper time for ERM implementation.

Regarding the ERM-uncertain category, the concentrations of air pollutants continuously decreased from D − 1 to D + 1, apart from those of SO_2_. The concentration of SO_2_ showed no significant changes over the time period (Fig. 2d). In the ERM-miss category, the PM_2.5_ and CO concentrations did not increase meaningfully from D − 1 to D0 (Fig. 2a and b) and the NO_2_ and SO_2_ concentrations continuously decreased for the 3 days (Fig. 2c and d). In all categories, regardless of ERM implementation, the concentrations of all air pollutants decreased after D0. Air pollutants can be regulated by various factors, such as meteorological conditions, domestic emissions, and the transboundary transport of air pollutants. Consideration of these factors is required to confirm the effectiveness of ERM in reducing PM_2.5_.

### Meteorology

**Fig. 3:**
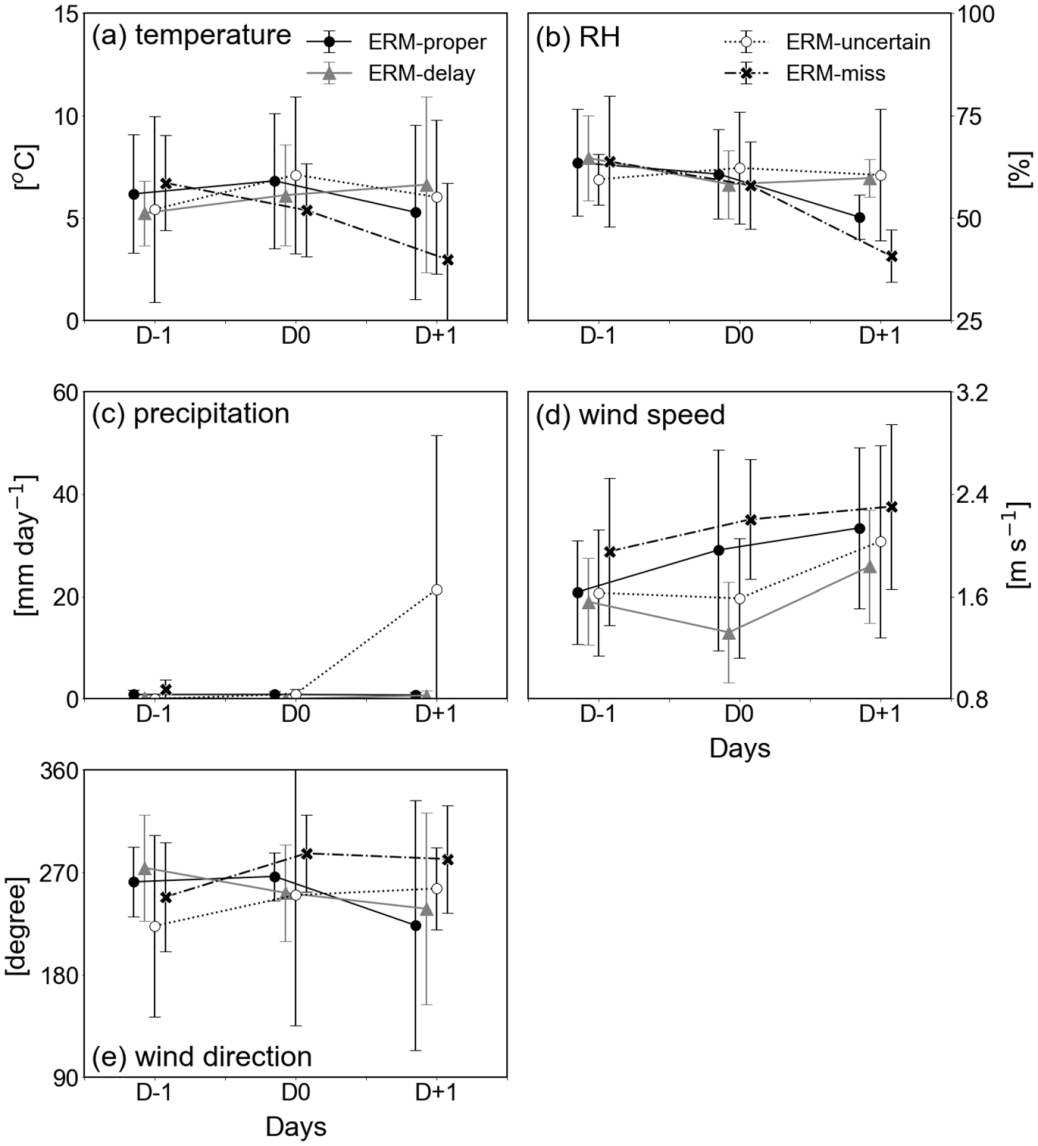
Meteorological variables of **a **temperature, **b **relative humidity (RH), **c **precipitation, **d **wind speed, and **e **wind direction, as in Fig. 2

Changes in air pollutants are principally modulated by meteorological conditions. It is well known that abundant precipitation may wash suspended pollutants out on the surface (Choi et al., 2008), and strong winds may ventilate the pollutants into the atmosphere or surrounding regions (Jung et al., 2019; Kim et al., 2017c; Lee et al., 2011). The surface air temperature affects the resuspension and formation of secondary aerosols (Lee et al., 2021; Seo et al., 2020). RH is associated with PM washout effects on precipitation and chemical processes for secondary aerosol formation (Kim et al., 2017d; Nguyen et al., 2017). Variations in four major atmospheric variables, namely surface air temperature, RH, precipitation, and wind speed, were investigated (Fig. 3). All variables showed a much larger difference between D0 and D + 1 than between D − 1 and D0. This indicates that the deteriorated air quality may be primarily resolved by changes in atmospheric circulation. Discrepancies among the four categories were largest at D + 1.

For 3-day surface air temperature variations (Fig. 3a), the discrepancies in the four categories were irrelevant except for D + 1, which was approximately 7 ℃ (3 ℃) in the ERM-delay (ERM-miss) category. RH also showed large divergence, particularly at D + 1 (Fig. 3b). The difference in the RH between the ERM-delay and ERM-miss categories was 20%. The warm and humid conditions in the ERM-delay category, which are favorable for the secondary formation of aerosols, appeared to be related to the persistence of high PM_2.5_ concentrations (see Fig. 1b). However, precipitation was not effective in reducing pollutants in the atmosphere for 3 days in all categories (Fig. 3c). Apart from one heavy precipitation event (64 mm) at D + 1 in the ERM-delay category, the precipitation amounted to less than 2 mm on all days and in all categories. Winds from the west and southwest were strongest at D + 1 in all categories (Fig. 3d and e), indicating the effective dispersion of air pollutants into the troposphere or adjacent regions in association with active ventilation. This suggests that PM_2.5_ concentrations could be decreased by meteorological conditions at D + 1, regardless of ERM implementation.

### Transportation

**Fig. 4:**
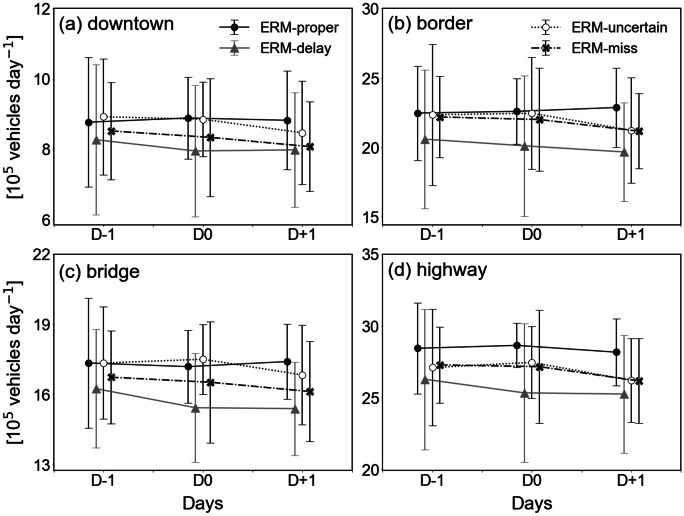
Traffic volumes in **a **downtown, **b **border, **c **bridge, and **d **highway, as in Fig. 2

The ERM law was primarily enacted to control the emission of air pollutants within Seoul City and the surrounding regions. Vehicle traffic is known to be the chief source of air pollutants in Seoul, which has no massive factories or industrial complexes. Figure 4 illustrates the variations in traffic volumes before and after ERM implementation in several locations in Seoul. It is natural to expect reduced traffic at D0 to be a successful execution of ERM; however, Fig. 4 seems to contradict this expectation. Traffic volumes at D0 did not decrease compared to D − 1 and D + 1 in four city areas: downtown, border, bridge, and highway. This is plausible considering that traffic regulations are compulsory only for employees of public and administrative institutions during ERM implementation. The number of public officials in Seoul was only 50,000 out of 9.55 million people (https://kosis.kr) and there were no significant differences between the categories. Traffic volume decreased slightly in the ERM-delay category, but it was difficult to declare a meaningful reduction considering the differing days of the week and the social situation on the day of ERM implementation.

### Power generation

**Fig. 5:**
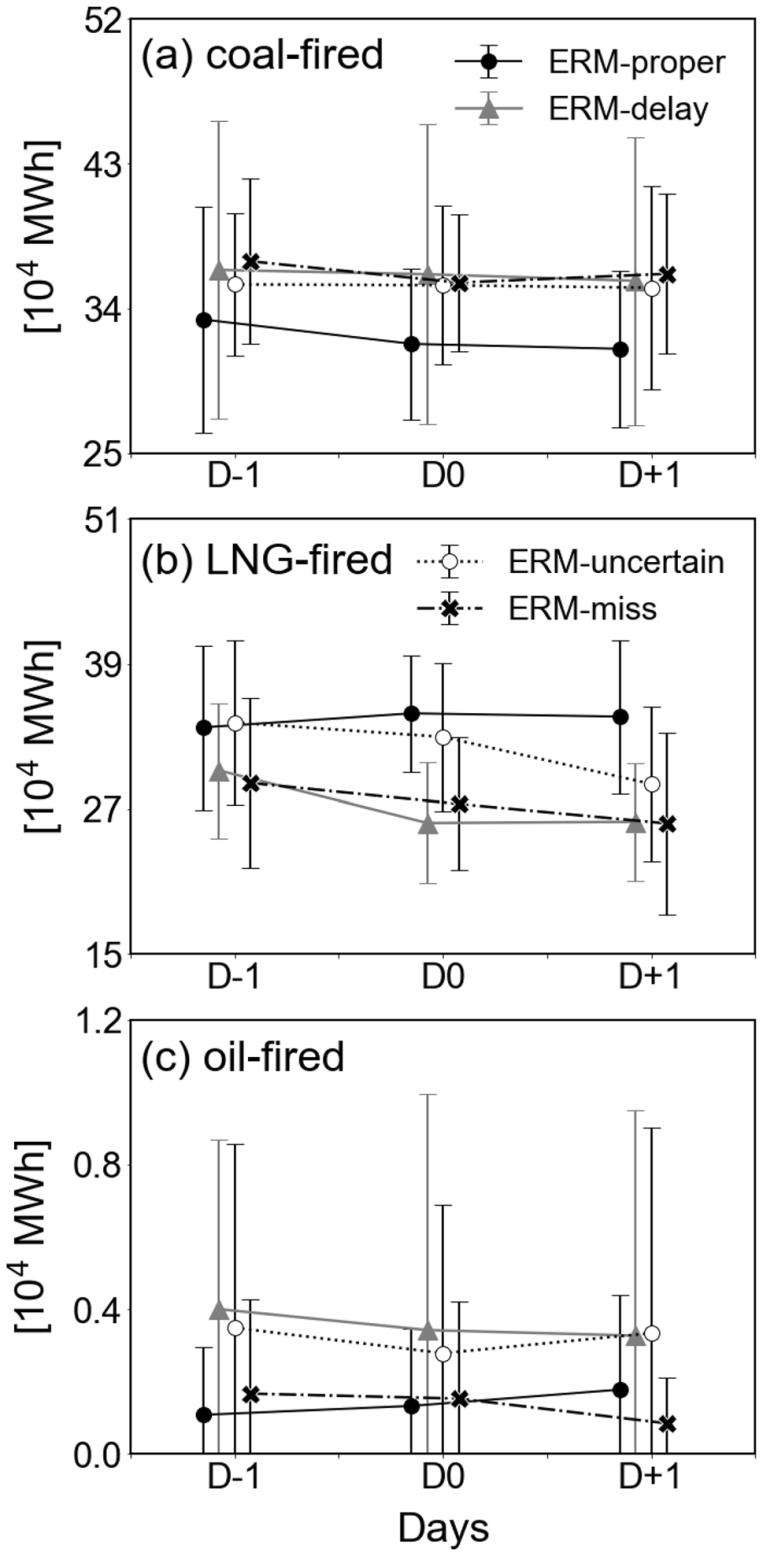
Electricity generation from thermal power plants, **a **coal-fired, **b **LNG-fired, and **c **oil-fired, as in Fig. 2

Power generation is a major contributor to air pollution in Korea, emitting a substantial amount of pollutants compared to residential and other sectors (Kim et al., 2016b, 2017a, b; Park et al., 2020). When ERM is executed, there is a constraint to reduce the thermal power generation, especially from coal and oil fuels. The effect of ERM should be considered, because power generation is managed by the Korean government. Figure 5 shows the 3-day variations in thermal power generation by coal-, LNG-, and oil-fired power plants. Overall, the temporal variations were not significant because it is difficult to restart a power plant within the short period of 1 or 2 days after its shutdown. Coal- and oil-fired power generation was lowest in the ERM-proper category (Fig. 5a and c). Specifically, the power generation from oil fuel was less than half of the value in the ERM-delay and ERM-uncertain categories (Fig. 5c). In LNG-fired power generation, the electricity amounts in the ERM-proper and ERM-uncertain categories were relatively high compared to those in the other two categories (Fig. 5b). The air pollutants emitted from LNG fuel are smaller than those emitted from coal or oil; thus, the government has stipulated the use of LNG. Although the operation of thermal power plants should be regulated in ERM-implemented categories, there was no significant change, except in the ERM-proper category. This indicates that ERM was not effectively implemented, resulting in no noticeable reduction in air pollutant emissions associated with PM_2.5_ concentrations.

### Large manufacturing company emissions

**Fig. 6:**
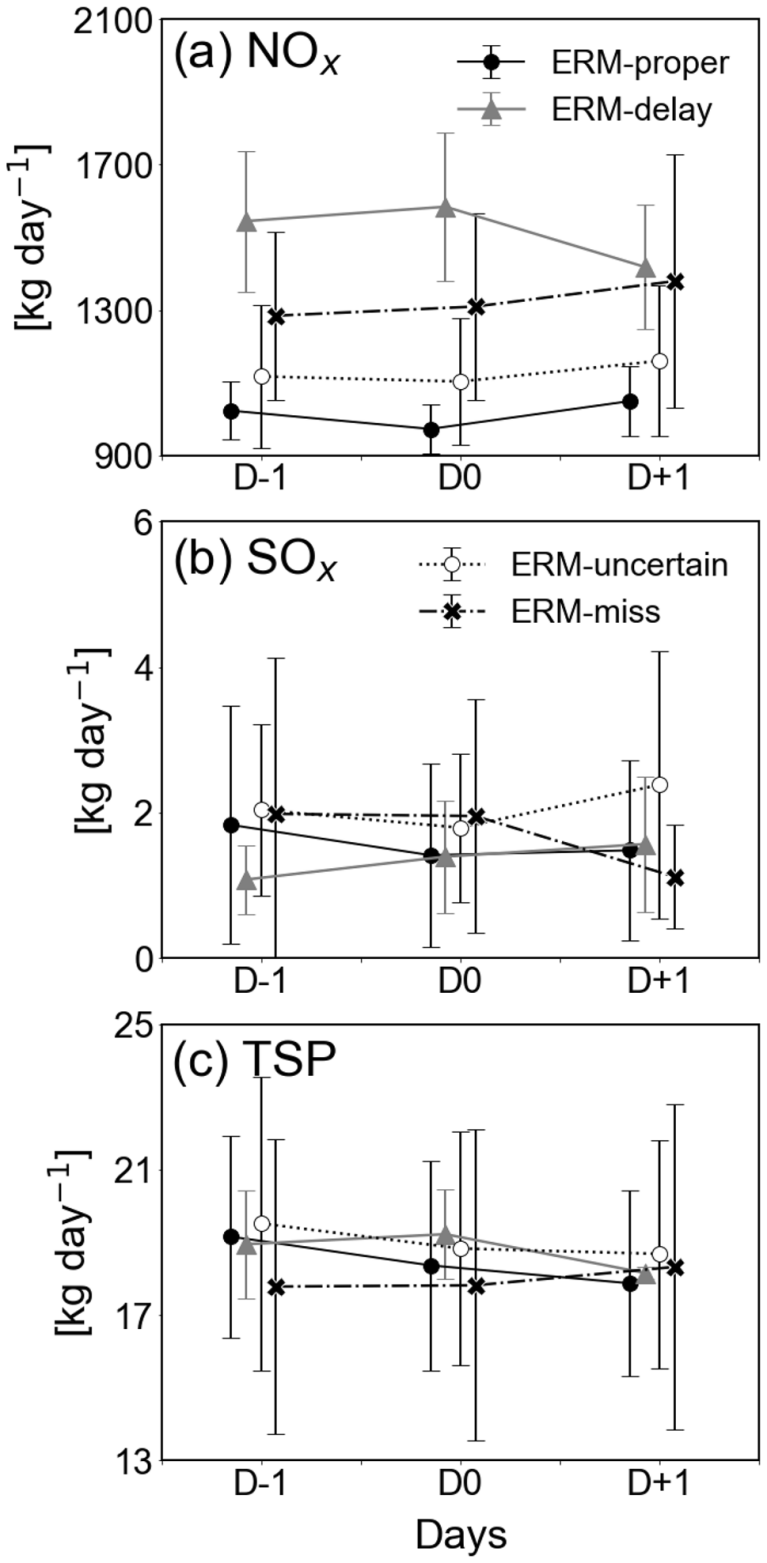
Emissions from large manufacturing companies, **a **NO_x_, **b **SO_x_, and **c **TSP, as in Fig. 2

Because emissions from large amounts of fossil fuel use cannot be ignored, the operation of large manufacturing companies is regulated during ERM implementation. This is likely to be successfully managed, because the locations are fixed, unlike vehicular transportation. Variations in emissions, such as NO_x_, SO_x_, and TSP, from large manufacturing companies were investigated before and after ERM implementation in Seoul (Fig. 6). All emissions showed a decrease on D0, the day of the ERM execution. For the variation in NO_x_ (Fig. 6a), the emissions decreased by 40–170 kg only in the three ERM-implemented categories; which is 3–10% of the total NO_x_ emissions. By contrast, there was no reduction in the ERM-miss category. The amounts of SO_x_ were the smallest of the three emissions, ranging from 0 to 2 kg, and decreased by 0.3 kg in the ERM-implemented categories (Fig. 6b). The lower SO_x_ emissions are attributed to the smaller number of emitting companies, as mentioned in the data section. The variation in TSP emissions was similar to that of NO_x_ emissions (Fig. 6c) and remained at approximately 18 kg for 3 days. After the ERM implementation, the reduction in TSP was about 0.7–3 kg (3–15%), except in the ERM-miss category. Overall, emissions showed a weak decrease in large manufacturing companies in the ERM regulatory sectors, which is consistent with the variations in air pollutants in Fig. 2. However, in Seoul, we could not determine the extent to which the reduction in emissions from large manufacturing companies affects PM_2.5_.

### Regional source apportionment

**Fig. 7:**
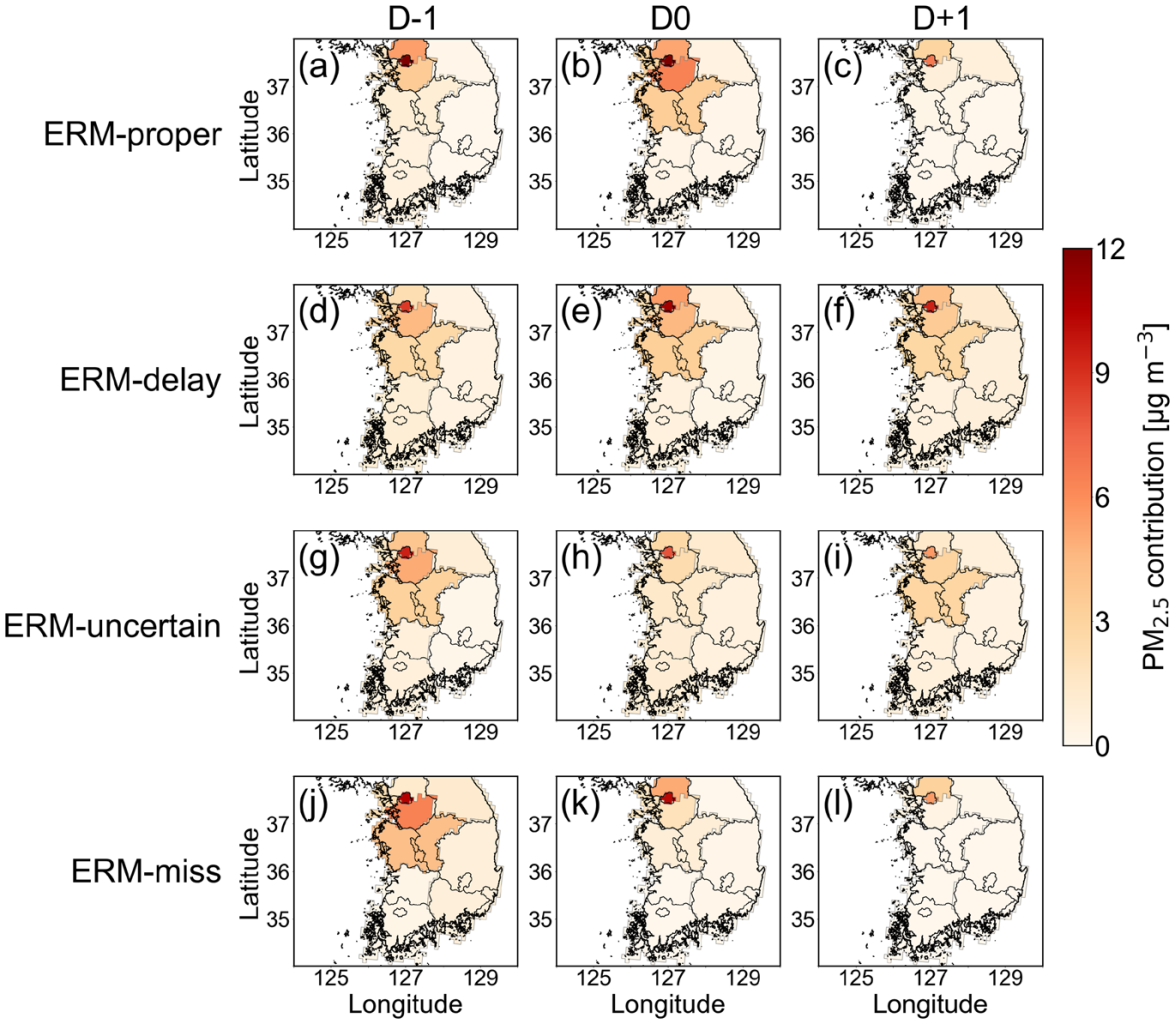
Domestic CAMx-PSAT source contributions to PM_2.5_ concentrations in Seoul for the four ERM categories, ERM-proper, ERM-delay, ERM-uncertain, and ERM-miss at D − 1, D0, and D + 1

Air quality deterioration in Seoul is largely influenced by the inflow of air pollutants from neighboring domestic regions and specifically, China (Lee et al., 2013; Oh et al., 2020). The transboundary transport of pollutants may accelerate the conditions that satisfy ERM implementation. While the amounts of pollutants transported from outside Seoul cannot be calculated by analyzing synoptic observations, the numerical model used can quantitatively simulate the delivered amount and source location. Figure 7 shows the contribution of the domestic CAMx-PSAT source to the PM_2.5_ concentrations in Seoul for the four ERM categories 3 days before and after ERM implementation. In all categories and days, Seoul had the large local contribution in the range of 5–14 μg m^−3^, accounting for 12–21% of the PM_2.5_ concentration in Seoul. The domestic contribution was also high on many D − 1 and D0 days, especially in northern Gyeonggi, southern Gyeonggi, and Chungcheong provinces, at approximately 4, 5, and 3 μg m^−3^, respectively (Fig. 7a, b, d, e, g, and j); those in other regions were less than 2 μg m^−3^. At D + 1, when the ERM implementation conditions were resolved, the contributions from outside Seoul did not exceed 3 μg m^−3^, except in the ERM-delay category (Fig. 7c, i, and l).

In the ERM-proper category (Fig. 7a-c), the domestic contribution of PM_2.5_ concentration was the highest in Seoul and northern Gyeonggi province at D − 1. The increase in contribution expanded to the southern Gyeonggi and Chungcheong regions located in southern Seoul at D0. In the ERM-delay category, the contributions of PM_2.5_ from Seoul and its neighboring regions (northern Gyeonggi, southern Gyeonggi, and Chungcheong provinces) were also high from D − 1 to D + 1 (Fig. 7d-f). In particular, the contribution from northern Gyeonggi rapidly increased at D0, indicating the transport of PM_2.5_, from the region located in northern Seoul. Within the ERM-uncertain category (Fig. 7g-i), the domestic contributions of PM_2.5_ were high in Seoul, southern Gyeonggi, and Chungcheong at D − 1. However, the contributions from these three regions decreased rapidly at D0. Similar changes in the domestic contributions also appeared in the ERM-miss category (Fig. 7j-l). The contributions at D0 slightly increased in northern Gyeonggi but decreased considerably in the neighboring regions of Seoul. The contribution from Seoul decreased to less than 2 μg m^−3^ on the day of ERM implementation in the other three categories, whereas there was no change in the ERM-miss category. This confirms the effect of ERM on the reduction in PM_2.5_ concentration in Seoul. However, the decrease in the regions neighboring Seoul was 6 − 8 μg m^−3^, which contributed to the reduction in the domestic contribution to PM_2.5_ in Seoul.

**Fig. 8:**
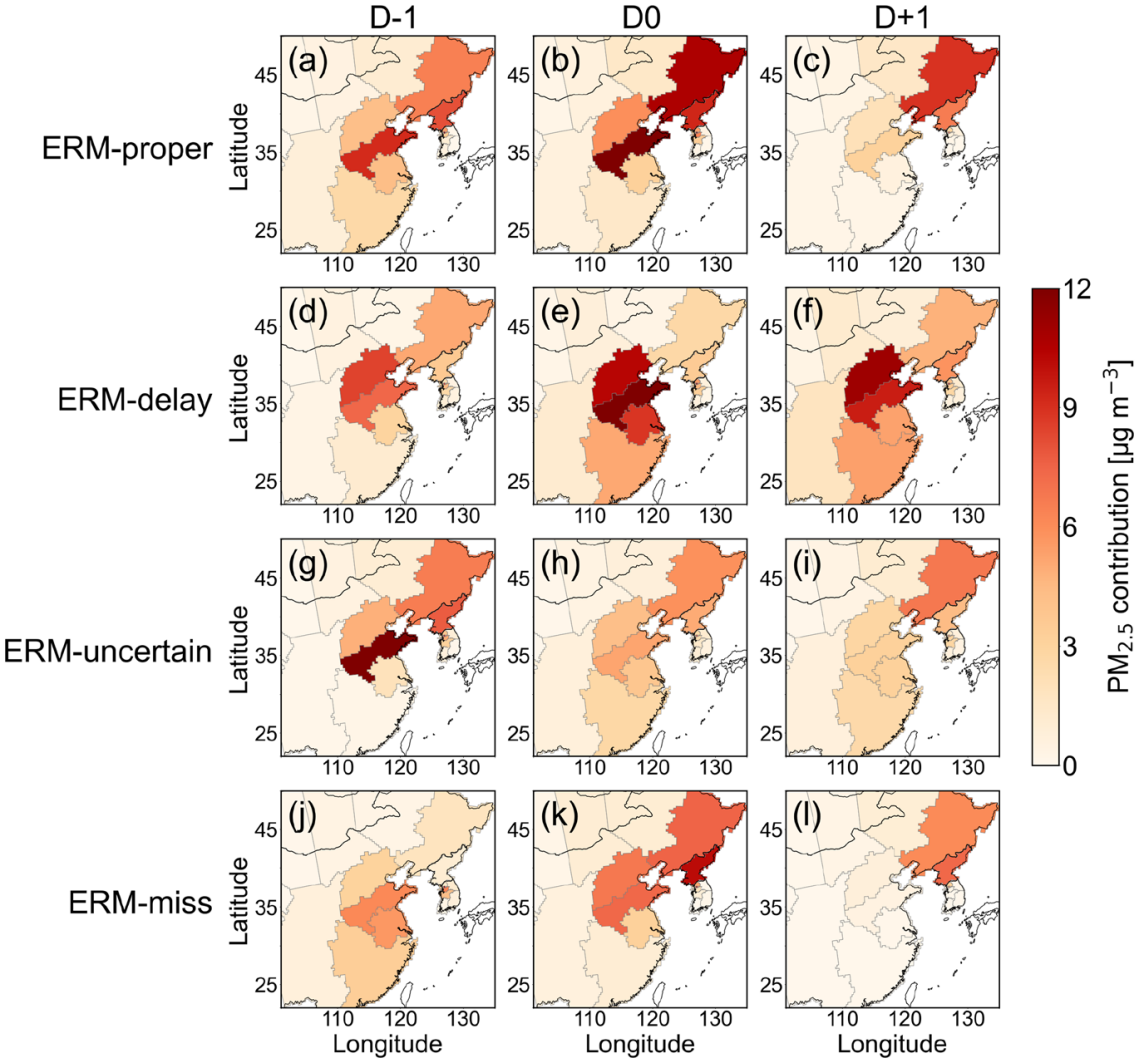
Foreign CAMx-PSAT source contributions to PM_2.5_ concentrations in Seoul for the four ERM categories, ERM-proper, ERM-delay, ERM-uncertain, and ERM-miss at D − 1, D0, and D + 1

The fact that transboundary transport from China has aggravated air quality in Korea is no longer a matter of debate (e.g., Chang et al., 2021; Lee et al., 2013, 2021; Oh et al., 2020); this is supported by the source contributions from China in Fig. 8, which shows that foreign sources contributed to Seoul PM_2.5_. For all four categories, the foreign contribution to PM_2.5_ concentration was within the range of 16–47 μg m^−3^ (45–70%) over the entire period. Specifically, except for D − 1 in the ERM-miss category, foreign contributions accounted for more than 60%. This means that most of the air pollutants in Seoul came from foreign countries, specifically from China. The total foreign contribution was highest at D − 1 or D0, similar to the domestic contribution. In all categories, the foreign contributions of these 2 days were consistently high, at 6–16 μg m^−3^ in Shandong province. This also increased to a similar level in northeastern China, Hebei province, and North Korea, which are located clockwise from Shandong province, especially on D0. The contributions of these four regions were 77–93% of all foreign contributions in all categories throughout the period. In the ERM-proper category (Fig. 8a-c), the foreign contributions were highest in northeastern China, Shandong province, and North Korea at D − 1. These contributions increased further at D0 and began to decrease from D + 1, with the largest decrease in contribution in Shandong province. The foreign contributions in the ERM-delay category (Fig. 8d−f) were high for Hebei and Shandong provinces, and the contributions of these two regions increased rapidly on D0. Additionally, there was an increase in the Yangtze River Delta. In the ERM-proper and ERM-delay categories, both domestic and foreign contributions increased, so the ERM implementation in Seoul might be meaningless.

Within the ERM-uncertain category (Fig. 8g-i), foreign contributions were highest in Shandong at D − 1. However, the next day, the contribution of this region rapidly decreased by more than half, and that of the other regions no longer increased. Compared with the increase in foreign contributions at D0 in the other three categories, the decrease in the ERM-uncertain category was noteworthy (Fig. 8j-l). In addition, on the same day, domestic contributions decreased, resulting in a decrease in PM_2.5_ concentration in Seoul. On the days in the ERM-miss category, foreign contributions rapidly increased in northeastern China, Hebei province, Shandong province, and North Korea. However, the total foreign contributions were relatively lower than those in the ERM-proper and ERM-delay categories. In addition, domestic contributions in the regions neighboring Seoul also decreased considerably at D0; thus, the PM_2.5_ concentration did not increase significantly. This suggests the necessity of strengthening the implementation of reduction measures in regions in Seoul to reduce the concentration of PM_2.5_. However, the most important aspect is the reduction in air pollutants in foreign regions.

## Conclusion

The present study analyzed PM_2.5_, major air pollutants, and some meteorological variables for 33 days of ERM implementation and 6 days without ERM implementation (i.e., ERM-miss). The 33 days of ERM implementation were further divided into proper (ERM-proper: 11 days), late (ERM-delay: 15 days), and uncertain (ERM-uncertain: 7 days) implementations. The concentration of PM_2.5_, except in the ERM-delay category, decreased below 50 μg m^−3^ after one ERM implementation. There was also no increase in the concentrations of other air pollutants such as CO, NO_2_, and SO_2_. These variations were the same for the ERM-miss category. High concentrations, which lasted for days in the ERM-delay category, were affected by relatively warm and wet weather conditions. Based upon the analysis results of the various ERM regulatory sectors, only large manufacturing companies’ emissions showed a weak decrease after ERM implementation. Considering the results of regional contribution analysis using PSAT in CAMx, these decreases contributed to reducing PM_2.5_ concentrations by less than 2 μg m^−3^ in Seoul. Nevertheless, foreign contributions to PM_2.5_ concentrations were mostly over 60% in all days and categories. The high foreign contributions lasted longest in the ERM-delay category, while they dropped promptly in the ERM-uncertain category. These results strongly indicate that air pollutants carried by transboundary transportation play a more important role than ERM implementation for the control of PM_2.5_ concentration in Seoul.

The possible influence of weather conditions on PM_2.5_ concentration in Seoul was also identified in this study. It was confirmed that relatively cold and dry conditions with strong wind speed affect the reduction in PM_2.5_ concentrations in all categories except for ERM-delay, but the effect of ERM implementation may overlap. Such weather conditions were maintained regardless of ERM implementation, while contributions from Seoul decreased only in the ERM-implemented category; therefore, the effect of ERM could be separated. In addition, the decrease in domestic contributions from Chungcheong, northern, and southern Gyeonggi provinces in the ERM-uncertain and ERM-miss categories was more effective in reducing PM_2.5_ concentrations in Seoul. Because the possibility of ERM implementation cannot be ignored in these areas, further analysis of the ERM effect in the neighboring regions of Seoul is required. The results of this study demonstrate that more intensive ERM implementation and close monitoring are needed in Seoul and its surrounding areas in the future. Moreover, these findings could provide a basis for promoting cooperation in the implementation of regular control policies between countries to improve the air quality in Korea.

## Data Availability

The datasets generated during and/or analyzed during the current study are available from the corresponding author on reasonable request.
